# Nanobody-based CTLA4 inhibitors for immune checkpoint blockade therapy of canine cancer patients

**DOI:** 10.1038/s41598-021-00325-3

**Published:** 2021-10-21

**Authors:** Jonathan Marable, Damien Ruiz, Anil K. Jaiswal, Ritankar Bhattacharya, Robert Pantazes, Payal Agarwal, Amol S. Suryawanshi, Deepa Bedi, Amarjit Mishra, Bruce F. Smith, Maninder Sandey

**Affiliations:** 1 Department of Pathobiology, College of Veterinary Medicine, Auburn University, 169 Green Hall, Auburn, AL 36840 USA; 2 Scott Ritchey Research Center, College of Veterinary Medicine, Auburn University, Auburn, AL USA; 3 Department of Chemical Engineering, Samuel Ginn College of Engineering, Auburn University, Auburn, AL USA; 4 Biomedical Sciences, Tuskegee University, Tuskegee, AL USA

**Keywords:** Cancer models, Antibody therapy

## Abstract

Cancer is the leading cause of death in the geriatric dog population. Currently, the use of immune checkpoint inhibitors (ICIs) such as anti-CTLA4 antibodies has markedly improved the prognosis of several cancers in their advanced stages. However, ICIs targeting CTLA4 blockade to treat canine cancer patients are yet to define. In this study, we sought to develop, characterize and assess whether chimeric heavy chain only antibodies (cHcAbs) against CTLA4 are viable therapeutic candidates for the treatment of canine cancers. Anti-CTLA4 nanobodies (Nbs) were identified from a yeast nanobody (Nb) library using magnetic-assisted cell sorting (MACS) and flow cytometry. cHcAbs were engineered by genetically fusing the DNA sequences coding for anti-CTLA4 Nbs with the Fc domain of the subclass B of canine IgG. Recombinant cHcAbs were purified from ExpiCHO-S cells. Stable cell lines expressing canine CTLA4 and FcγRI were used to elucidate the binding ability and specificity of cHcAbs. PBMCs isolated from healthy dogs were used to evaluate the ability of cHcAbs to activate canine PBMCs (cPBMCs). Novel Nbs were identified using the extracellular domain of canine CTLA4 protein to screen a fully synthetic yeast nanobody library. Purified Nbs bind specifically to natïve canine CTLA4. We report that chimeric HcAbs, which were engineered by fusing the anti-CTLA4 Nbs and Fc region of subclass B of canine IgG, were half the size of a conventional mAb and formed dimers. The chimeric HcAbs specifically binds both with canine CTLA4 and Fcγ receptors. As the binding of Nbs overlapped with the MYPPPY motif of canine CTLA4, these Nbs were expected to sterically disrupt the interaction of canine CTLA4 to B-7s. Like their human counterpart, canine CTLA4 was expressed on helper T cells and a small subset of cytotoxic T cells. Canine Tregs also constitutively expressed CTLA4, and stimulation with PMA/Ionomycin dramatically increased expression of CTLA4 on the cell surface. Stimulation of cPBMCs in the presence of agonistic anti-CD3 Ab and cHcAb6 significantly increased the expression of IFN-γ as compared to the isotype control. This study identifies a novel nanobody-based CTLA4 inhibitor for the treatment of canine cancer patients.

## Introduction

The development of immune checkpoint inhibitors (ICIs) has revolutionized the field of cancer immunotherapy. Recently, several ICIs have been approved by the US Food and Drug Administration (FDA) for the treatment of a wide array of cancer types^1–7^. Since ICIs provide sustained therapeutic responses over time, and they are now considered as the standard line of treatment and care for several malignancies. In normal physiological conditions, immune checkpoint molecules provide a balance between pro-inflammatory and anti-inflammatory signaling. In addition, immune checkpoint molecules are critical for maintaining self-tolerance, minimizing collateral tissue damage, and developing immunity against pathogens^8^. Tumor cells disrupt this balance by activating inhibitory immune checkpoints to create an immunosuppressive milieu, which favors immune evasion and tumor growth^8^. To date, antagonistic antibodies targeting three different molecules, including cytotoxic T lymphocyte-associated protein-4 (CTLA-4; ipilimumab), programmed cell death protein-1 (PD-1; nivolumab), or programmed cell death ligand 1 (PD-L1; atezolizumab) have been approved for use in humans^1–7^.

CTLA4 is a critical immunoregulatory molecule, which downregulates T-cell activation and promotes clonal anergy^9,10^. Naïve T cells require two important signals during their initial priming for optimum activation and clonal expansion^11^. The first signal is provided by the binding of T cell receptor (TCR) to peptide-major histocompatibility complexes (MHCs) on antigen-presenting cells (APCs). The engagement of CD28 to B7-1 (CD80) or B7-2 (CD86) gives the “second” stimulatory signal^11^. The CD28-mediated positive costimulatory signal is essential for T-cell activation, proliferation, and IL-2 production and thus is critical to induce an antigen-specific T cell response. Conversely, CTLA4, also expressed on T cells, competitively inhibits the binding of B7-1 or B7-2 to CD28 and blocks the positive costimulatory signal^11,12^. Blocking of CD28-B7 interaction by CTLA4 prevents TCR downstream phosphorylation cascade, attenuating T cell activation, thereby, induces T cell tolerance^6–8^. In addition, tumor-infiltrating regulatory T cells (Tregs) express CTLA4 at high levels and utilize it to establish an immunosuppressive milieu in the tumor microenvironment (TME)^12–15^. Therefore, blockade of CTLA4 with antagonistic mAbs (ipilimumab) increases the survival time and causes remission in subsets of human melanoma patients^2,5^. The blockade of CTLA4-B7 interaction by ipilimumab allows unrestrained CD28-mediated positive stimulation and activation of cytotoxic T cell responses^16–18^.

However, the therapeutic benefits of ipilimumab are often limited to a small subset of cancer patients, and cancer patients who initially respond usually develop acquired resistance. Multiple approaches, including combinatorial immunotherapy with ICIs targeting divergent signaling pathways, could augment positive outcomes and extend these benefits for several tumor types^6,7^. However, the development and optimization of different combinations of immune checkpoint blockade (ICB) drugs are hampered by the lack of an excellent preclinical animal model. Although mouse models of human cancer are invaluable to understand the mechanistic concepts of immunotherapy; however, they often fail to recapitulate the complexity of the tumor stages manifested in human disease. This caveat with the absence of spontaneous cancers in mouse models creates another layer of complexities and challenges for clinical translation studies.

Naturally occurring cancer in pet dogs is the leading cause of death in the geriatric canine population^19,20^. Pet dogs develop several cancers, including malignant melanoma, lymphoma, mammary carcinoma, gliomas, and osteosarcoma, which closely resemble the clinical manifestation, metastasis, recurrence, genetic predisposition, and response patterns or resistance-to-treatment seen in human cancer patients^21–26^. Pet dogs also share our environment and thus are exposed to the same environmental risk factors. The tumor initiation, progression, metastasis, and development of immune response in dogs closely resemble human disease. Therefore, naturally occurring cancer in pet dogs is an ideal animal model for studying complex immune interactions during cancer treatment^27–29^. Various studies have shown that the CTLA-4 plays a similar role in canine cancer^30^. However, ICIs for CTLA4 blockade are not commercially available for canine cancer patients due to the lack of species-specific mAbs.

Here, we developed nanobody (Nb)-based ICIs to elicit CTLA4 blockade and treat canine cancer patients. Nanobodies (Nbs), representing the variable fragments (VHH) of camelid heavy-chain-only antibodies (HcAbs), bind to antigens in the absence of a light chain^14^. Nbs are small in size (~ 15 kDa) and bind to antigens with high specificity and affinity like conventional mAbs^30,31,33^. Nbs or Nb-based molecules are in different stages of development for cancer diagnosis and therapy^31–34^. In this study, we have identified several canine-specific anti-CTLA4 Nbs from a fully synthetic yeast Nb library using magnetic-assisted cell sorting (MACS) and assessed their activity and therapeutic potential. Taken together, we believe our report is the first of its kind to develop and characterize nanobody-based canine-specific ICIs to overcome the primary perceived limitation of understating canine cancer. Furthermore, our report provides a critical proof-of-concept for developing anti-human CTLA4 Nbs in future clinical studies.

## Methods

All animal procedures, including collection of blood samples from healthy donors (colony animals), were reviewed and approved by the Auburn University Institutional Animal Care and Use Committee (IACUC). All methods are reported in accordance with ARRIVE guidelines for the reporting of animal experiments. All methods were performed in accordance with relevant guidelines and regulations.

### Identification of anti-canine CTLA4 Nbs

Nbs targeting canine CTLA4 were identified using a yeast surface display platform following the published protocol^35^. The recombinant canine CTLA4 protein (Sino Biologicals) was labeled either with Alexa fluor-647 (AF 647) or biotin using microscale protein labeling kits (ThermoFisher). The labeled canine CTLA4 protein was used for biopanning, and CTLA4 binders were enriched by magnetic-assisted cell sorting (MACS) using anti-AF647 or anti-biotin microbeads (Miltenyi). For each round of MACS selection, yeast was grown for 48–72 h in galactose supplemented tryptophan dropout media (Sigma) to induce Nb expression. Initially, 2.5 × 10^9^ induced yeast cells were washed, resuspended, and incubated at 4 °C for 30 min with anti-Alexa Fluor 647 microbeads in selection buffer (20 mM HEPES, pH 7.5, 150 mM sodium chloride, 0.1% (w/v) ovalbumin, 1 mM EDTA). The yeast cells were then passed through the LD column (Miltenyi) to remove yeast-expressing Nbs that bound nonspecifically to magnetic beads. After pre-clearing, yeast cells were incubated with labeled canine CTLA-4 protein (1 µM final concentration) at 4 °C for 30 min. Yeast cells were then incubated with anti-AF647 microbeads at 4 °C for 30 min and passed through the LS column to enrich for yeast-expressing Nbs specific to canine CTLA4 protein. The enriched yeast was collected, grown, induced, and used for the second round of MACS selection using biotin-labeled canine CTLA4 protein. A total of three rounds of MACS selection were performed. We also used successively lower concentrations (1 µM for 1st round; 500 nM for 2nd round; and 75 nM for 3rd round) of CTLA4 protein to enrich high-affinity binders. After the 3rd round of MACS selection, yeast cells were grown, induced, and labeled with AF647-labeled canine CTLA4 protein, anti-HA AF488 antibody, and propidium iodide. Yeast cells positive for AF647, AF488, and negative for propidium iodide were sorted into single clones in 96-well plates using a flow sorter (Daiko).

### Purification of Nbs

Three yeast clones (cNb6, cNb13 and cNb17) with a strong affinity for canine CTLA-4 protein were selected for in-depth characterization^35^. The DNA sequences coding for Nbs were amplified by polymerase chain reaction (PCR) and cloned into a periplasmic expression vector pET22b. The Nb genes were tagged with 6xHis and Strep Tag II on the C-terminus and expressed in BL21 (DE3) *E. coli*. For each purification, *E. coli* were cultured in 500 mL of terrific broth (MP Biosciences) and induced after reaching OD_600_ 0.6–0.8 with 1 mM Isopropyl β-d-1-thiogalactopyranoside (IPTG, Zymo Research) at 25 °C for 16–18 h. Induced cells were pelleted and resuspended in 25 mL of TSE buffer (0.2 M Tris–HCL, pH 8, 200 g/L sucrose, 0.5 mM EDTA). 50 mL of cold, sterile water was added to the cells and stirred for 45 min @ 4 °C to provide an osmotic shock to release periplasmic Nbs. The periplasmic fraction containing the tagged Nbs was centrifuged at 16,000×*g* for 10 min @ 4 °C. The supernatant was collected and dialyzed against 2× PBS buffer overnight @ 4 °C using a 3 kDa cutoff dialysis cassette (ThermoFisher). The dialyzed proteins were centrifuged at 10,000×*g* for 15 min @ 4 °C, and the supernatant was loaded onto a 5 mL StrepTrap column (GE Healthcare) using an AKTA Explorer (GE Healthcare). The column was washed with 10 column volumes (CV) of 2× PBS buffer and bound Nbs were eluted with 2× PBS buffer containing 2.5 mM desthiobiotin. The eluted Nbs were concentrated using a protein concentrator (Cytiva) with a molecular weight cutoff (MWCO) of 5 kDa. The concentrated Nbs were applied to a superdex 200 10/300 GL column to further purify Nbs using size exclusion chromatography. The recombinant protein was concentrated to 1.0 mg/mL using 3 kDa protein concentrators (Pierce), filter sterilized (0.2 µM), snap-frozen in liquid nitrogen, and stored at − 80 °C. Purified Nbs were analyzed by SDS-PAGE to confirm purity.

### Expression and purification of chimeric heavy chain only antibodies (cHcAbs)

The nucleotide sequences coding for cNb6 were fused in silico with the hinge and Fc domain of subclass B of canine IgG^36^. A secretion signal from the V-J2-C region of the mouse Ig Kappa-chain followed by a Strep II Tag were also cloned at the N-terminus for efficient secretion and purification, respectively. The fused sequence was synthesized (Gene Universal) and cloned into a mammalian expression vector pCDNA3.1/Hygro (+). The recombinant plasmid was transiently transfected into ExpiCHO-S cells using ExpiFectamine CHO transfection kit (ThermoFisher). The ExpiCHO-S cells and conditioned media were harvested after 7 days. Following harvest, the supernatant containing the cHcAbs was clarified by centrifugation at 4000×*g* for 30 min. The conditioned media was diluted (1:2) with binding buffer (20 mM sodium phosphate) and applied to HiTrap protein A HP column (Cytiva) using AKTA explorer. The resin was washed with 10 column volumes of wash buffer (10 mM sodium phosphate [pH 7.0]). Proteins were eluted with 100 mM sodium citrate (pH 3.0) directly into 1 M Tris–Hcl (pH 8; 0.25 mL/mL elution). The purified cHcAbs were buffer exchanged into 1X PBS and applied to Superdex200 inrease10/300 GL column for size exclusion chromatography. The purified cHcAbs were concentrated using 10 kDa protein concentrators (Pierce) to a final concentration of 1.0 mg/mL, filter-sterilized (0.2 µM), snap-frozen in liquid nitrogen, and stored at − 80 °C. Endotoxin levels were measured using the ToxinSensor Chromogenic LAL Endotoxin Assay Kit (Genscript), and all cHcAbs contained less than five endotoxin units per milligram of protein.

### Purification of canine peripheral blood mononuclear cells

30 mL of peripheral blood was collected from three healthy dogs in heparinized tubes. The blood sample was diluted 1:1 with Dulbecco's phosphate buffer saline (DPBS). Two Sepmate PBMC isolation tubes were filled with 15 mL of histopaque. The diluted blood sample was carefully layered over the Sepmate insert. The layered blood was centrifuged at 800×*g* for 10 min. The PBMCs layer was carefully collected and diluted with DPBS in a new 15 mL conical tube. The cell suspension was mixed well and centrifuged at 800×*g* for 10 min. The supernatant was discarded, and the cell pellet was resuspended and washed again with DPBS. After the supernatant was discarded, the cells were resuspended in complete RPMI-1640 media containing 10% fetal bovine serum (FBS) plus penicillin (200 U/mL) and streptomycin (100 µg/mL).

### SDS-PAGE and western blotting

The expression and purity of eluted Nbs and chimeric HcAbs were determined by sodium dodecyl sulfate–polyacrylamide gel electrophoresis (SDS-PAGE). The SDS-PAGE gels were stained with GelCode Blue Stain Reagent to assess the purity of the purified nanobodies, cHcAb6 and cHcAb13. For western blotting, conditioned media containing cHcAbs was separated under reducing (beta-mercaptoethanol) and non-reducing conditions and transferred to the nitrocellulose membrane. The cHcAb6 band was detected with anti-Strep Tag II and rabbit anti-canine IgG Fc antibodies (Novus Biologicals). Secondary antibodies conjugated with IRDye680RD (LI-COR Biosciences) were used to detect the primary antibodies. Blots were scanned on LI-COR Odyssey 9120 digital scanning system.

### Cell culture and in vitro cell line generation

Stable cell lines were generated to assess the binding of anti-CTLA4 Nbs and cHcAbs. Briefly, MDCK cells were transfected with recombinant plasmids expressing canine FcγRI or CTLA4 using Lipofectamine 3000 (Life Technologies) according to the manufacturer’s directions. Cells were selected with hygromycin and flow-sorted to obtain single-cell clones. Clones expressing a high level of CTLA4 and FcγRI were used for flow cytometry. MDCK, MDCK cell expressing FcγRI (MDCK-FcγRI) or CTLA4 (MDCK-CTLA4) were maintained in DMEM media supplemented with 10% fetal bovine serum (FBS) at 37 °C in 5% CO_2_.

### Flow cytometry

MDCK, MDCK-FcγRI and MDCK-CTLA4 cells were trypsinized and washed with FACS buffer (1× PBS containing 1% bovine serum albumin (BSA), 2 mM EDTA, 0.02% sodium azide, and HEPES). Cells were incubated in blocking buffer (PBS containing 4% goat or rabbit serum, 1% BSA, 0.02% sodium azide) and stained with anti-CTLA4 Nbs and cHcAbs. Bound Nbs or cHcAbs were detected by using anti-His 647 or anti-canine IgG Fc 647 or 750 secondary antibodies and analyzed by flow cytometry. Canine PBMC were cultured overnight in RPMI media. PBMCs were stimulated with various concentrations (50, 100 and 150 ng/mL) of PMA and Ionomycin (1 µM/mL final concentration) for 6 h. Stimulated PBMCs were blocked and stained with anti-CTLA4 Nbs and cHcAbs. Cells were also stained with anti-CD3FITC (clone CA17.2A12), anti-CD4RPE (clone YKIX 302.9), and anti-CD8 647 (clone YCATE55.9) antibodies to characterize subpopulations expressing canine CTLA4. For intracellular FOXP3 staining, cells were fixed and permeabilized using the fix & perm cell fixation and cell permeabilization kit from Life Technologies and stained with FOXP3 monoclonal antibody (FJK-16s [ThermoFisher]).

### In vitro functional assays

All animal procedures, including collection of blood samples from healthy dogs, were reviewed and approved by the Auburn University IACUC. CPBMCs isolated from three healthy dogs were stimulated with anti-CD3 antibody (1 μg/mL) in the presence or absence of cHcAb6 (100 nM). After 3 days, total RNA was isolated, reverse-transcribed, and quantified for IFN-γ mRNA levels using TaqMan assays (Cf02623316_m1). All assays were performed in triplicates, and HPRT1 (Cf02690456_g1) was used as endogenous control. Canine IgG was used as isotype control.

### Protein structure modeling

The complementarity determining regions (CDRs) of the nanobodies cNb6, cNb13, and cNb17 were determined from their genetic sequences using the multiple sequence alignment tool Clustal Omega^37^, EMBL-EBI^38^. The structures of the three anti-CTLA4 Nbs, the extracellular domain of canine CTLA4, and the entirety of canine CTLA4 were predicted from their genetic sequences using I-Tasser^39^, Robetta^40^, and trRosetta^41^ (15 total predicted structures). As the computational methods are predictions, multiple methods were utilized to provide insight into whether there was consensus or differences among varying approaches. The ZDOCK server^42^ was used to globally dock the individual Nbs predicted by each method with the corresponding canine CTLA4 extracellular domains. 30 results were generated for each of the protein prediction methods (10 per Nb per method, 90 total predicted complexes). These complexes were observed using the molecular visualization tool UCSF Chimera^43^.

### Statistical analysis

All experiments were performed in triplicates. The quantitative PCR data was analyzed using a two-samples t-test. All statistical analysis was performed with SAS. All statistical tests were two-sided, and a P-value of < 0.05 was regarded as statically significant.

## Results

### Selection of anti-canine CTLA4 Nbs from the library

**Figure 1:**
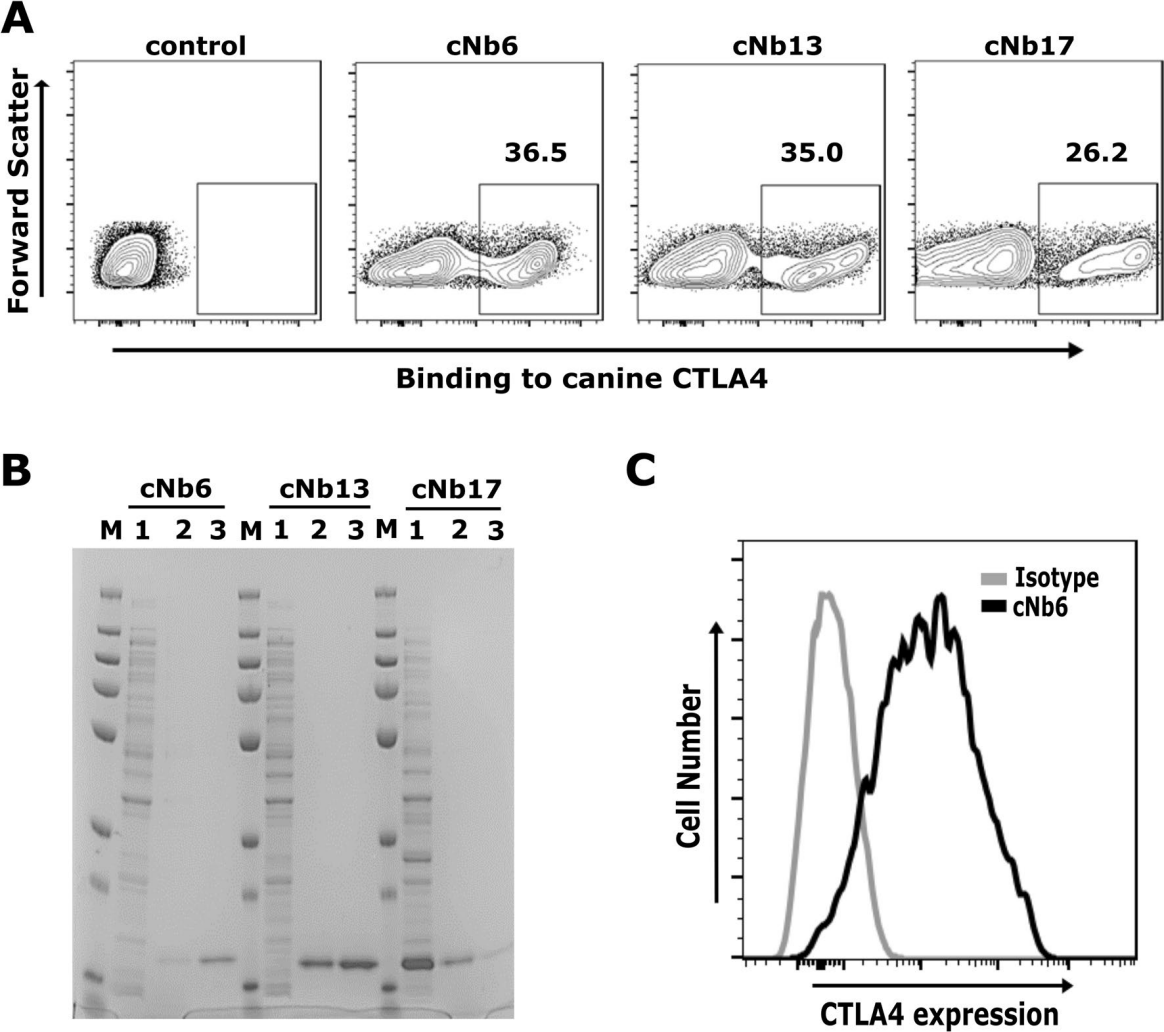
Discovery of nanobodies targeting canine CTLA4. (**A**) Single yeast clones bind to canine CTLA4. After three rounds of MACS selection, the enriched yeast nanobody library was sorted by FACS to obtain single yeast clones. Single yeast clones were induced with galactose to express nanobody on their cell surface and labeled with 100 nM of AF647-labeled canine CTLA4 protein. Three yeast clones (cNb6, cNb13 and cNb17) showed excellent binding to AF647-labeled canine CTLA4 protein. Noninduced yeast cells (cNb6) do not bind to AF647-labeled canine CTLA4 protein. (**B**) Purity of nanobodies assessed by SDS-PAGE. DNA sequences coding cNb6, cNb13 and cNb17 were amplified and cloned into a pET22b vector. Nbs were expressed and purified in *E. coli* BL21 (DE3) cells by affinity (StrepTrap) and size-exclusion chromatography (Superdex 10/300 GL). Purified nanobodies were resolved on SDS-PAGE gel and stained with GelCode Blue Stain Reagent. M—protein ladder, 1—unpurified protein, 2 and 3—1st and 2nd elute of purified Nbs. (**C**) Binding of cNb6 to cells expressing canine CTLA4 demonstrated by flow cytometry. MDCK cells transiently expressing CTLA4 were stained with cNb6, washed, and bound cNb6 was detected with anti-His-647 by flow cytometry. A non-specific Nb was used as an isotype control.

We were able to screen Nbs targeting canine CTLA4 from a fully synthetic nanobody library^35^. Nbs with high affinity were identified using a successive lower concentration of recombinant CTLA4 protein and three rounds of MACS selection. The percentage of CTLA4 binding yeast cells progressively increased from < 1 to ~ 50% after three rounds of MACS. To isolate single yeast clones, AF647^+^, HA488^+^ and Propidium Iodide^−^ yeast cells were sorted from an enriched Nb library using fluorescence-activated cell sorting (FACS). As expected, most single yeast clones showed excellent binding to the canine CTLA4 protein (Fig. 1A). Based on maximum binding to AF647 labeled CTLA4 protein, we selected three yeast clones (cNb6, cNb13, and cNb17) for further in-depth characterization. DNA sequences coding for these clones were amplified and cloned into a periplasmic expression pET-22b^(+)^ vector for purification. Nbs were expressed in *E. coli* (DE3) and purified from the periplasmic fraction using affinity and size-exclusion chromatography. We found high levels of Nbs expression both in the periplasm as well as in the media. As shown in Fig. 1B, we confirmed the purity of Nbs by SDS-PAGE. A recombinant plasmid was constructed to transfect and transiently express canine CTLA4 protein. Madin-Derby canine kidney (MDCK) cells expressing CTLA4 protein were stained with anti-CTLA4 Nbs. The anti-CTLA4 Nbs (cNb6, cNb13, and cNb17) bind specifically to MDCK cells expressing canine CTLA4 protein, while binding was absent on untransfected MDCK cells (Fig. 1C). Taken together, these results suggests an effective approach of identifying canine-specific anti-CTLA4 Nbs (cNb6, cNb13, and cNb17) from a fully synthetic Nb library.

### Anti-CTLA4 Nbs bind to a conserved MYPPPY motif of canine CTLA4:

**Figure 2:**
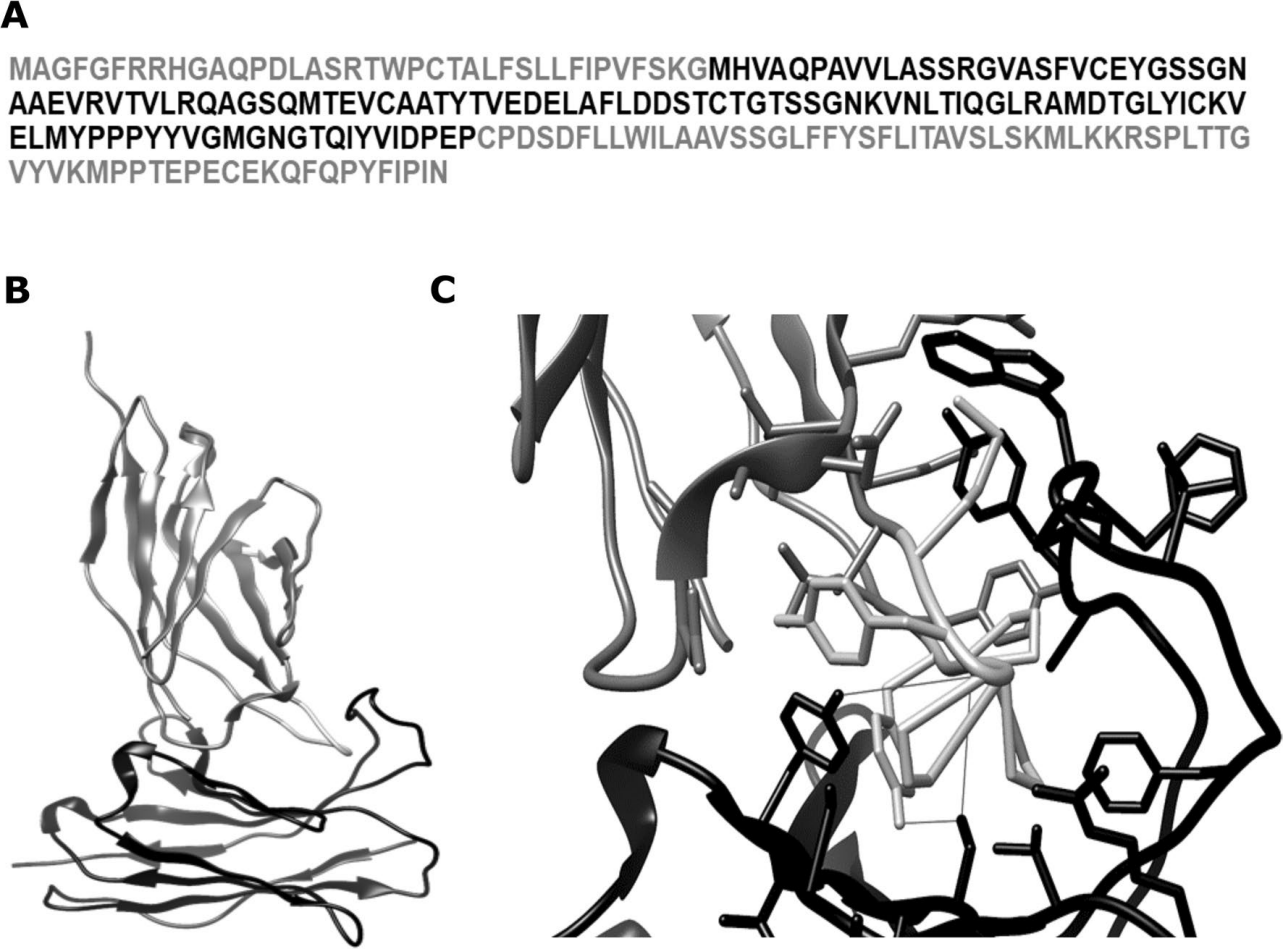
Anti-CTLA4 nanobodies binding to the MYPPPY epitope of canine CTLA4. (**A**) The FASTA sequence of canine CTLA4, where the conserved modeled domain has been highlighted in black and the rest in light gray. (**B**) A globally docked complex of anti-CTLA4 nanobody cNb6 (black) and canine CTLA4 (dim gray) with the MYPPPY epitope (dark gray) interacting with the nanobody CDRs. (**C**) A detailed view of the interacting molecules in (**B**) with intermolecular hydrogen bonds (fine gray straight lines) between Ser-Pro/Ser-Tyr/Tyr-Pro residues near the center of the image. Additionally, there are several intermolecular aromatic residue interactions around the target epitope, which represents hydrophobic interactions.

All structure prediction methods (i.e., I-Tasser, Robetta, and trRosetta) showed poor-quality predictions for the full canine CTLA4 sequence (Fig. 2A); however, they elicit high-confidence predictions for the extracellular domain. Since Nbs interact only with the extracellular domain, these structures were further used for docking calculations. Each structure prediction method also generated Nb structures with very high confidence scores. Of the 90 Nb/CTLA4 complexes predicted by the ZDOCK server, 85 showed Nb CDRs interacting with an MYPPPY epitope in CTLA4 (Fig. 2B,C) that is conserved among humans^44^, mice^45^, and dogs. The remaining five complexes each showed Nb/CTLA4 interactions that are unrealistic (i.e., interactions not mediated through the CDRs). All predicted complexes were provided as Supplemental Information. As shown in Fig. 2C, the intermolecular amino acid interactions for cNb6 were within the distance of 5 Å from the MYPPPY epitope. The epitope residues of each were predicted to participate in hydrophobic packing and hydrogen bond interactions with residues from CDRs 2 and 3 of cNb6. In addition to the conserved epitope, Tyr103 (the next amino acid in the C-terminal direction) were also predicted to form a hydrogen bond with the backbone of Nb CDR3. Collectively, these data show the binding ability of anti-CTLA4 Nbs to a conserved region of canine CTLA4.

### Engineering chimeric anti-CTLA4 canine heavy-chain-only antibodies (cHcAb)

**Figure 3:**
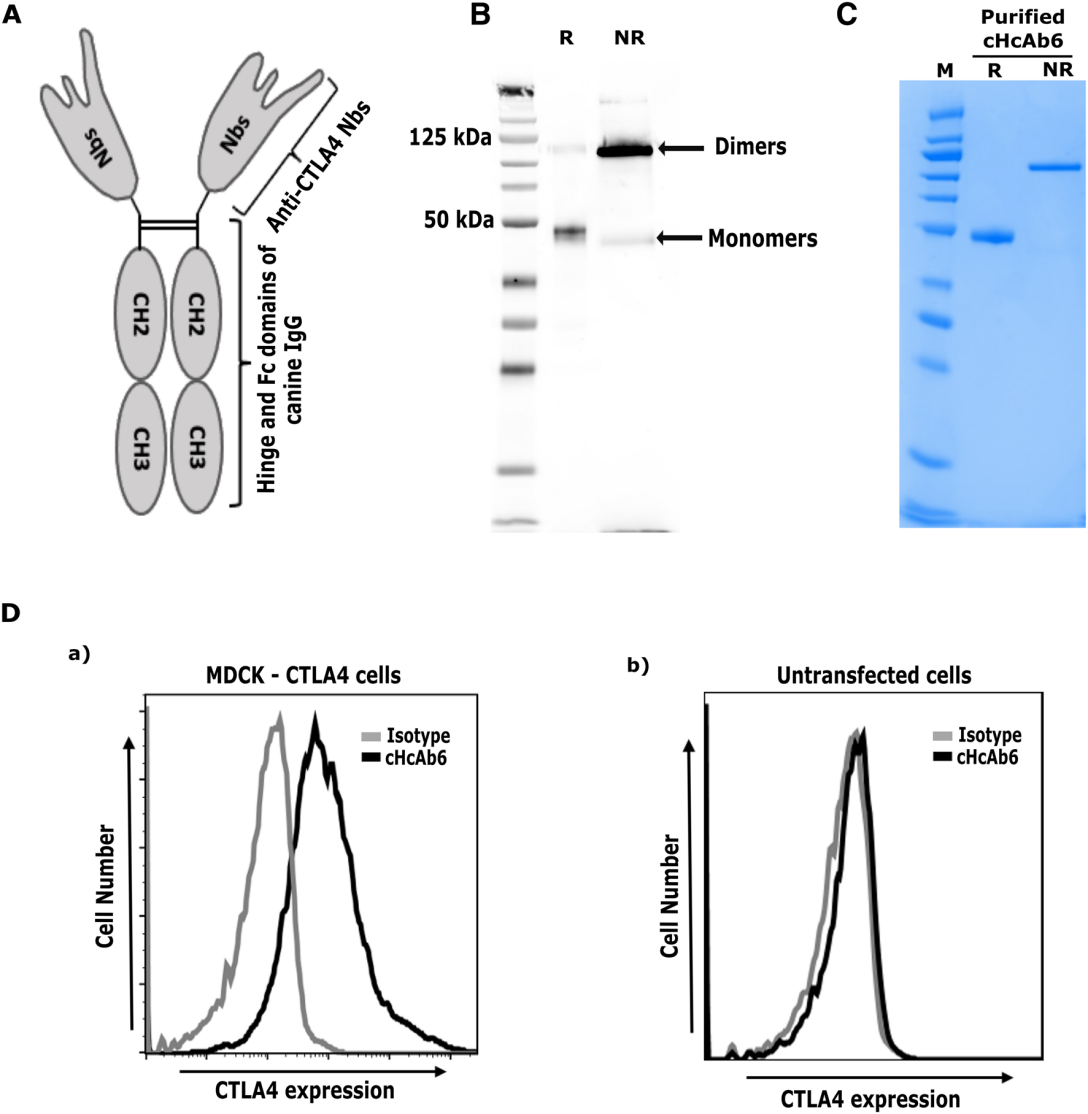
Development of Nb-based anti-CTLA4 heavy chain only antibody (HcAb). (**A**) Predicted structure of cHcAb6. The DNA sequence coding for the cNb6 was genetically fused to the hinge and Fc domains of canine IgG (subclass B). (**B**) The cHcAb6 form dimers as demonstrated by western blotting. The cHcAb6 was expected to form dimers via hinge and Fc domains of canine IgG. The cHcAb6 protein, expressed in ExpiCHO-S cells, was resolved under reducing and non-reducing conditions and detected by anti-IgG Fc antibody. The cHcAb6, as expected, forms dimers of ~ 83 kDa under non-reducing conditions. (R-reducing condition, NR-non reducing). (**C**) Purity of cHcAb6 assessed by SDS-PAGE. The cHcAb6 was expressed and purified from the ExpiCHO-S cells by affinity (Protein A) and size-exclusion chromatography. The purified cHcAb6 was resolved under reducing (R) and non-reducing (NR) condition and stained with GelCode Blue Stain. (**D**) Binding of cHcAb6 to cells expressing canine CTLA4 demonstrated by flow cytometry. MDCK cells transiently expressing CTLA4 were stained with cHcAb6, washed, and bound cHcAb6 was detected using anti-Fc-750 antibody by flow cytometry. cHcAb6 does not bind to untransfected cells.

Although the small size of Nbs allows for a better biodistribution and tissue penetration, this can be seen as a disadvantage for therapy due to rapid clearance from the bloodstream by renal elimination^46,47^. To overcome this limitation, we successfully engineered a chimeric cHcAb6 by genetically fusing the Fc domain of canine IgG (subclass B) with cNb6. cHcAb6 was actively secreted by ExpiCHO-S cells into the conditioned media due to the presence of the secretion signal of the V-J2-C region of the mouse Ig Kappa-chain at its N-terminus. cHcAb6 was successfully purified from the conditioned media by affinity chromatography using the protein A/G column (Fig. 3). Two peaks representing the monomeric and dimeric forms of the recombinant protein were eluted from these columns. The presence of the Fc domain was further confirmed by immunoblots using an anti-canine IgG Fc antibody. As shown in (Fig. 3B), cHcAb6 forms dimers under non-reducing conditions. The reduced form of the cHcAb6 migrates at the predicated monomeric molecular weight (MW) of 46 kDa, while the dimeric form was ~ 75 kDa. We also replaced the cNb6 sequence in the cHcAb6 with the DNA sequences coding for cNb13 to construct cHcAb13**.** cHcAb13 was similarly expressed and purified from transiently transfected ExpiCHO-S cells. Both cHcAb6 and cHcAb13 showed excellent binding to MDCK cells expressing canine CTLA4, while no binding was observed on untransfected MDCK cells (Fig. 3D).

### Chimeric HcAbs bind to cognate CTLA4

**Figure 4:**
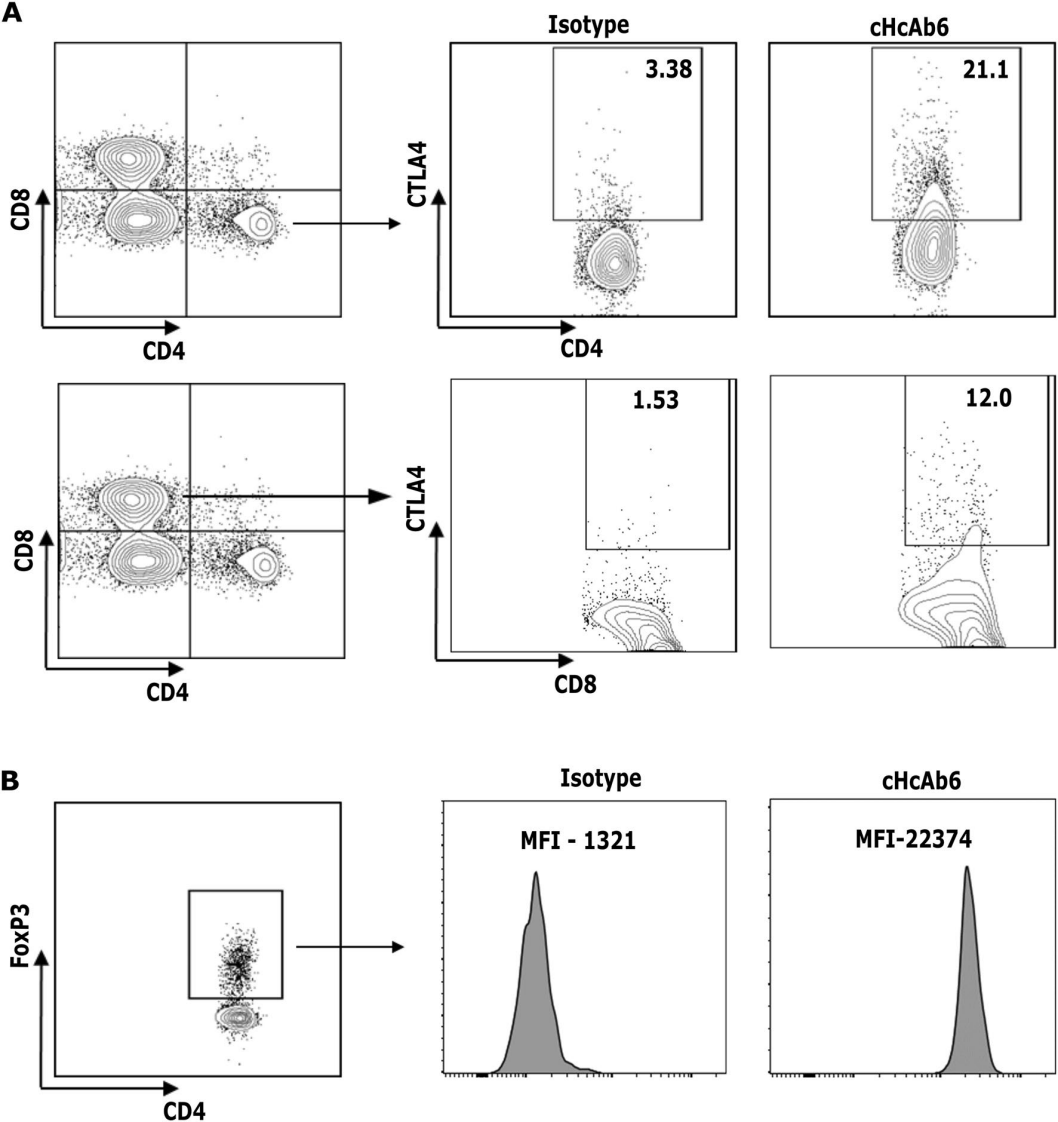
Canine Tregs constitutively express CTLA4. Activated cPBMCs were first stained with cHcAb6. The cHcAb6 stained PBMCs were fixed, permeabilized, and treated with anti-CD3, CD4, CD8, and FoxP3 antibodies. The bound cHcAb6 was detected using anti-canine IgG Fc-750 Ab. Canine IgG was used as an isotype control. (**A**) The CTLA4 was predominantly expressed on helper T cells and a small subset of cytotoxic T cells. (**B**) CTLA4 was constitutively expressed on Tregs. *MFI* Mean fluorescence intensity.

**Figure 5:**
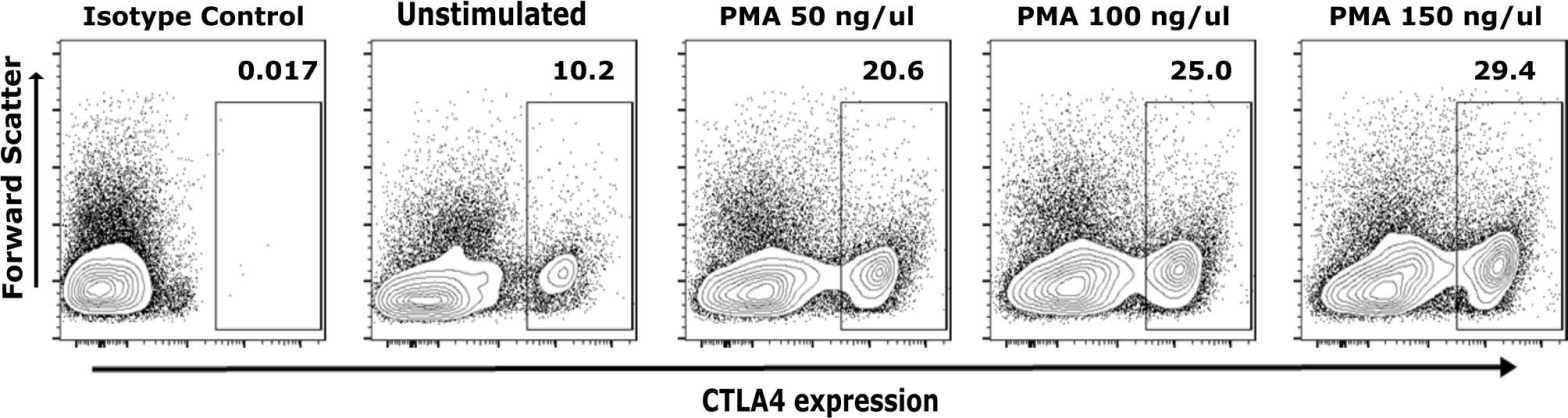
cHcAb6 binds to native CTLA4 on cPBMCs. CPBMCs were stimulated with PMA and Ionomycin for 8 h. Activated and control PBMCs were stained with cHcAb6 and analyzed by flow cytometry using anti-canine IgG Fc-750 Ab. Canine IgG was used as an isotype control. The CTLA4 expression was markedly increased after PMA/Ionomycin stimulation.

To further characterize the cHcAb6 and HcAb13 Nbs, studies were next performed for specific binding to native CTLA4 expressed on canine T cells. We purified canine peripheral blood mononuclear cells (PBMCs) from the blood collected from three healthy dogs. Both cHcAb6 & HcAb13 showed excellent binding to native CTLA4 expressed on canine T cells. CTLA4 was predominantly expressed on helper T cells (CD3^+^, CD4^+^); however, its expression was also detected in a small percentage of cytotoxic (CD3^+^, CD8^+^) T cells (Fig. 4A). The CTLA4 was constitutively expressed on regulatory (CD3^+^, CD4^+^ and Foxp3^+^) T cells (Fig. 4B). The expression of CTLA4 on the cell surface was markedly increased after PMA/Ionomycin stimulation (Fig. 5) as compared to unstimulated T cells.

### Chimeric HcAbs bind to canine FcγRI receptor and activate cPBMCs

**Figure 6:**
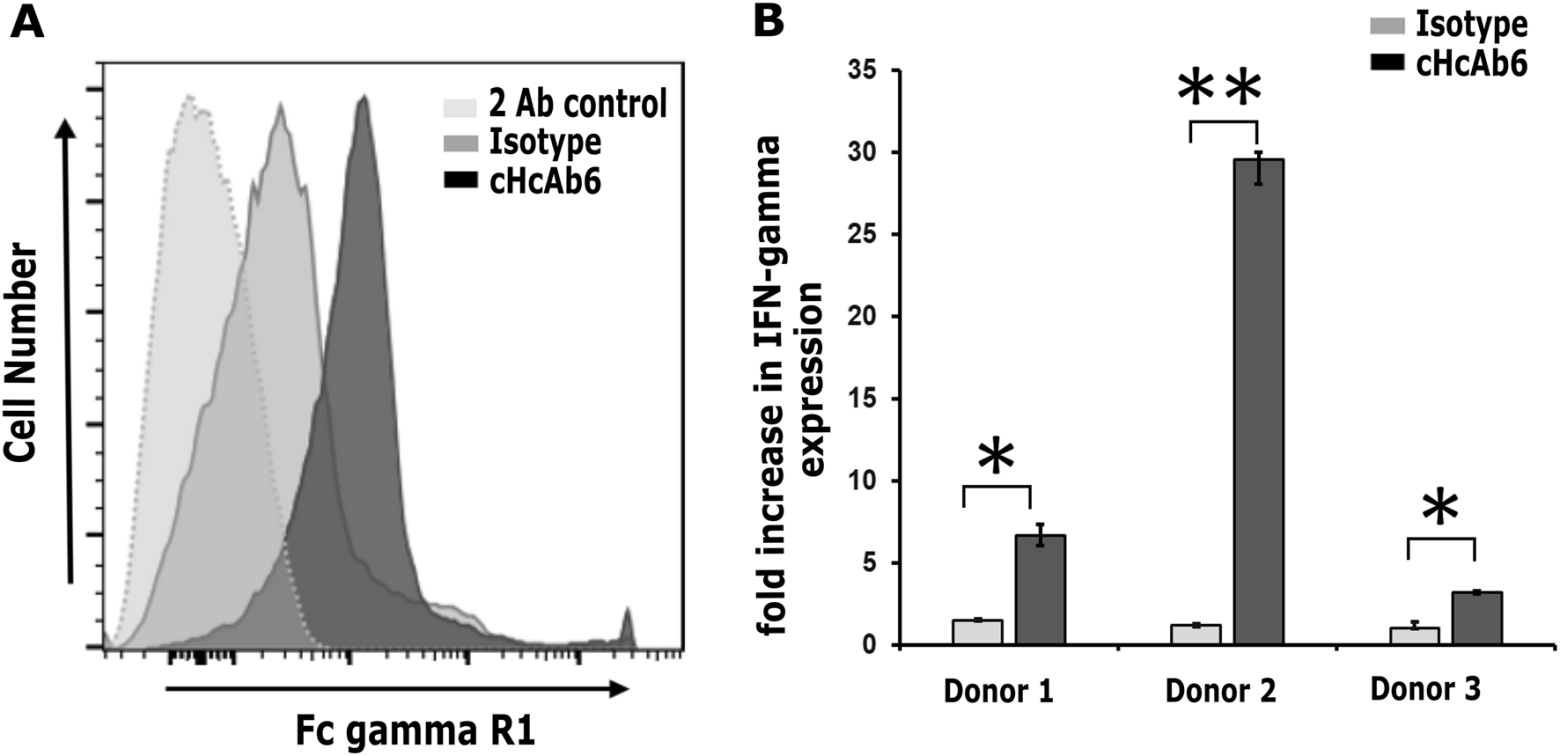
(**A**) cHcAb6 binds to cells expressing the canine FcγRI receptor. MDCK cells transiently expressing FcγRI were stained with cNb6, and bound cHcAb6 was detected using anti-Fc-750 antibody by flow cytometry. Canine IgG and cells stained with secondary Ab only were used as controls. (**B**) cHcAb6 induces IFN-γ expression from PBMCs after CD3 stimulation. CPBMCs were stimulated with 1 μg/mL of anti-CD3 antibody in the presence or absence of 100 nM of cHcAb6. After 72 h, IFN-γ expression was quantified using TaqMan assays. HRPT1 was used as endogenous control, and expression levels of IFN-γ were normalized to CD3 stimulated PBMCs. All experiments were performed in triplicates.

As binding of mAbs to Fc receptor is required for antibody-dependent cellular cytotoxicity (ADCC), we next evaluated the ability of cHcAbs to engage canine Fc receptor. The proper folding of the Fc domain was already confirmed as it binds to protein A/G. To ensure the binding of the Fc domain to the Fc receptor, we successfully cloned and expressed the canine FcγRI receptor (data not shown). Both cHcAb6 and cHcAb13 showed excellent binding to MDCK cells expressing canine FcγRI, while no binding was observed in untransfected MDCK cells (Fig. 6A). To further examine the functional activity of cHcAb6, cPBMCs were purified from three healthy dogs and stimulated sub-optimally with anti-CD3 mAb (1 μg/mL) in the presence or absence of cHcAb6. The IFN-γ expression levels were significantly increased in the presence of cHcAb6 compared to PBMCs treated with canine IgG (Fig. 6B). Taken together, these results confirmed that cHcAb6 was biologically functional and activates cPBMCs.

## Discussion

There are numerous benefits to the integration of pet dogs with naturally occurring cancers into immune-oncology research. Using this naturally occurring model, we can develop better therapeutics, explore pharmacokinetic/pharmacodynamics relationships, and carefully examine the clinical toxicity and bioactivity of novel immunotherapeutics^28,29^. Canine cancers arise spontaneously in the presence of an intact immune response and have similar pathological/molecular characteristics, transcriptome/immune profiles, and clinical presentation^22,23,27^. Like their human counterparts, canine patients also succumb to metastatic disease even after surgical amputation and adjuvant chemotherapy^24,25,29^. Moreover, immune cell populations in dogs and their role in tumor progression and metastasis are very similar to humans. In this study, we developed Nb-based CTLA4 inhibitors to treat canine cancer patients.

The anti-CTLA4 Nbs were identified from a yeast Nb library. The majority of the Nbs described to date are generated by immunizing camelids. However, this process is slow and highly expensive, posing a significant barrier to develop Nb-based immunotherapeutics for animal use We were able to identify Nb clones with efficient biologic activity by using only 300 µg of recombinant canine CTLA4 protein. Furthermore, the binding affinity of single yeast clones can be measured by flow cytometry. A binding curve can be plotted between mean fluorescence intensity and decreasing concentration of antigen. The nonlinear regression is then used to fit the curve using the one-site binding hyperbola and predict Kd values. The Kd values obtained by flow cytometry often correlate well with the affinities determined by bio‐layer interferometry (BLI)^48^.

Nb clones (cNb6, cNb13, and cNb17) identified in this study bind to the MYPPPY motif of canine CTLA4 protein^49^. This motif is conserved between dog, mouse, and human and is essential for interaction with the B7-1 (CD80) and B7-2 (CD86) ligands^18^. Thus, we expect these Nbs to sterically interfere and block the interaction of CD80 and CD86 to canine CTLA4. We were able to define the CTLA4 expression using our anti-CTLA4 Nbs in different subsets of T lymphocytes. Like their human counterparts, canine Tregs (CD4^+^, Foxp3^+^ T cells) also express CTLA4. CTLA4 is expressed at low levels on resting canine CD4^+^ T cells, which is rapidly upregulated after PMA/ionomycin stimulation.

Nb-based biologics inherently have a short plasma half-life due to their small size (15 kDa). Additionally, Nbs cannot trigger antibody-dependent cellular cytotoxicity (ADCC) or complement-dependent cytotoxicity (CDC) due to the lack of the Fc domain^49^. Fc-FcƳR interaction plays a central role in several murine models of CTLA4 blockade^35,49^. H11, an anti-mouse CTLA4 Nb, has minimal efficacy in vivo. The effectiveness of H11-based anti–CTLA-4 therapy can be restored via conjugating it to the Fc portion of murine IgG2a^49^. The role of the Fc-FcƳR interaction for the therapeutic efficacy of anti-CTLA4 therapy in human cancer patients is still not clear. The anti-CTLA4 antibody ipilimumab can deplete human Tregs in vitro; however, patients treated with ipilimumab do not definitively show Treg depletion. Thus, the Nb-based chimeric anti-CTLA4 HcAbs for this study were engineered via genetically fusing the Fc domain of the subclass B (functional analog to human IgG1) of canine IgG^36^. This subclass of canine IgG binds most tightly to C1q, FcƳRI, and FcƳRIII receptors^36^. We expect our chimeric HcAbs to block CTLA4 interaction with CD80 and CD80 via anti-CLTA4 Nbs while also provoking ADCC and CDC through the Fc domain. Consistent with this, we show that chimeric HcAb6 was able to induce IFN-γ expressions in CD3 + PBMCs in vitro, suggesting it can disrupt CTLA4 interactions with B-7s and/or facilitate Fc-dependent functions. In the future studies, we will confirm the ability of cHcAb6 to provoke ADCC and deplete Tregs.

The Nb-based ICIs approach in the canine model described in this report has several benefits. The novel CTLA4 inhibitors developed in this study have potential to treat canine cancer patients and to integrate them in immuno-oncology research. A similar approach can be used to develop ICIs targeting other inhibitory pathways (PD-1/PD-L1 and Tim-3) and co-stimulatory pathways (OX40 and 4-1BB). As clinical benefits are only observed in a small fraction of human cancer patients with monotherapies, the availability of novel canine-specific immunotherapeutics targeting other inhibitory pathways will allow optimization of doses/regimens of various combination therapies, predict their activity and/or potential toxicities before human trials. Moreover, as Nbs are monomeric and encoded by single piece of DNA, their genetic engineering to develop novel multimeric molecules is very easy. Two or more nanobodies can be cloned in tandem to make bispecific (recognizing two antigens) molecules for concomitant CTLA4 and PD1 blockade^50^. This integrative approach to develop novel immunotherapeutics in pets dogs with various cancer can benefit both humans and pets, as it will provide dogs with access to cutting-edge cancer treatments while ensuring that people are given treatments that are more likely to succeed.

## Conclusions

In summary, we successfully identified anti-CTLA4 Nbs that bind to the conserved MYPPPY motif and sterically disrupt the interaction of canine CTLA4 protein to B-7s. The chimeric HcAbs containing the Fc region of the canine IgG were able to interact with Fcγ receptors. These chimeric HcAbs were half the size of mAbs, bind specifically to canine Tregs and CD4^+^ helper T cells expressing CTLA4, and were able to induce expression of IFN-γ. Future studies employing pet dogs with various types and cancer stages are warranted to assess the therapeutic efficacy of chimeric HcAbs.

## Supplementary Information

Supplementary Information 1.

Supplementary Information 2.

## Data Availability

All data generated or analyzed during this study are included in this published article.

## Abbreviations

cHcAb: Chimeric heavy chain only antibody
CTLA4: Cytotoxic T lymphocyte antigen-4
DNA: Deoxyribonucleic acid
FACS: Fluorescence-activated cell sorting
HcAbs: Heavy chain only antibodies
ICB: Immune checkpoint blockade
ICIs: Immune checkpoint inhibitors
ICs: Immune checkpoints
IFN-γ: Interferon-γ
mAbs: Monoclonal antibodies
MACS: Magnetic-assisted cell sorting
MHC: Major histocompatibility complex
Nb: Nanobody
Nbs: Nanobodies
PD-L1: Programmed cell death ligand-1
PD-1: Programmed cell death protein-1
RNA: Ribonucleic acid
TCR: T-cell receptors
TME: Tumor microenvironment
cPBMCs: Canine peripheral blood mononuclear cells
