# Supplementary material for: Nanobody-based CTLA4 inhibitors for immune checkpoint blockade therapy of canine cancer patients
Source: Sci Rep. 2021 Oct 21;11:20763. doi: 10.1038/s41598-021-00325-3 (PMC8531395; doi:10.1038/s41598-021-00325-3)
Supplement: Supplementary file 2 — Supplementary Information 2. [file 41598_2021_325_MOESM2_ESM.docx]

**Additional File:**

**Supplementary Figure 1:** 90 Nb/CTLA4 complexes predicted by the ZDOCK server, 85 showed Nb CDRs interacting with an MYPPPY epitope in CTLA4. The remaining five complexes each showed Nb/CTLA4 interactions that are unrealistic (*i.e.* interactions not mediated through the CDRs). All predicted complexes were provided as Supplemental Information.

**Supplementary Figure 2:** The cHcAb6 and cHcAb13 form dimers as demonstrated by western blotting. The cHcAb6 and cHcAb13 were expected to form dimers via hinge and Fc domains of canine IgG. The cHcAb6 and cHcAb13 proteins, expressed in ExpiCHO-S cells, were resolved under reducing and non-reducing conditions and detected by anti-IgG Fc antibody. The cHcAb6 and cHcAb13 as expected, forms dimers of ~ 90 kDa under non-reducing conditions. (Lane M - Chameleon Duo Pre-stained Protein Ladder, Lane R- reducing condition & Lane NR - non reducing).


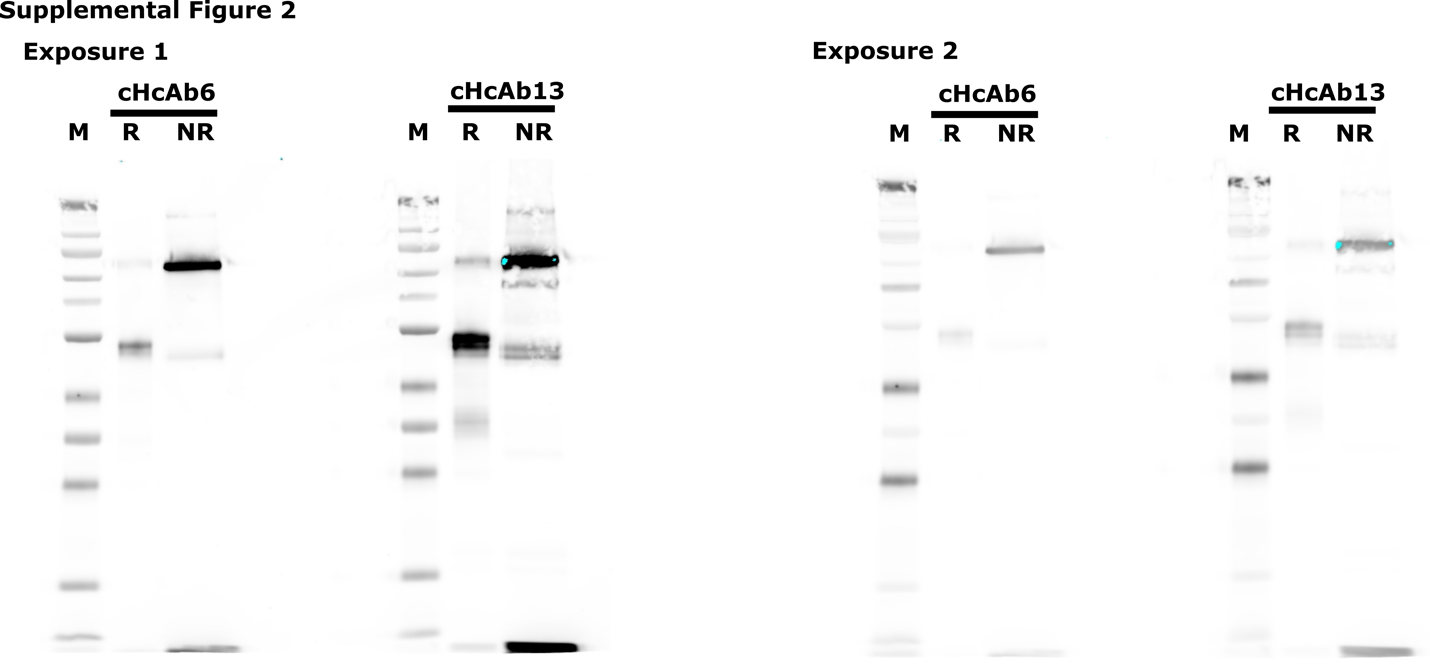


**Supplementary figure 3:** Purity of cHcAb6 and cHcAb13 assessed by SDS-PAGE. The cHcAb6 and cHcAb13 were expressed and purified from the ExpiCHO-S cells by affinity (Protein A) and size-exclusion chromatography. The purified cHcAb6 and cHcAb13 were resolved under reducing (R) and non-reducing (NR) condition and stained with GelCode Blue Stain. Lane M- Chameleon Duo Pre-stained Protein Ladder, Lane 1, 3, 5 & 7 - cHcAb6 and cHcAb13 resoloved under reducing condition & Lane 2, 4, 6 & 8 - cHcAb6 and cHcAb13 resoloved under non-reducing condition.

**
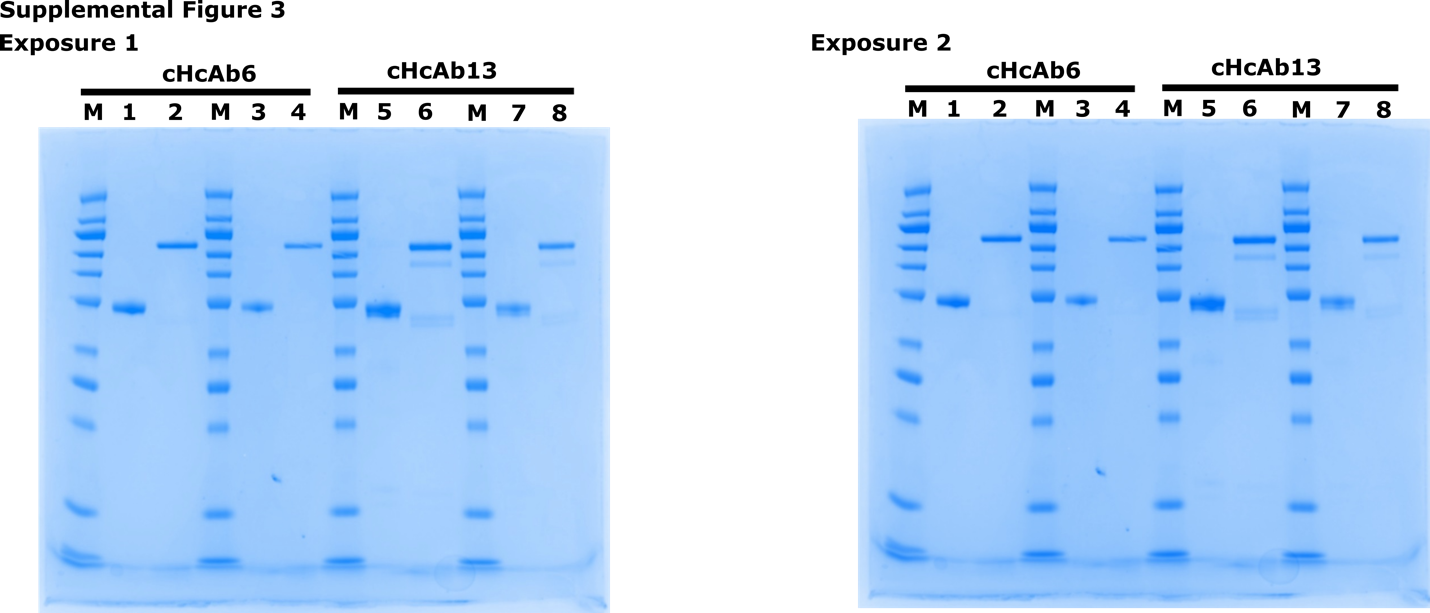
**
